# Small-Cell Lung Cancer in England: Trends in Survival and Chemotherapy Using the National Lung Cancer Audit

**DOI:** 10.1371/journal.pone.0089426

**Published:** 2014-02-21

**Authors:** Aamir Khakwani, Anna L. Rich, Laila J. Tata, Helen A. Powell, Rosamund A. Stanley, David R. Baldwin, Richard B. Hubbard

**Affiliations:** 1 Division of Epidemiology and Public Health, University of Nottingham, Nottingham, United Kingdom; 2 Department of Respiratory Medicine, Nottingham University Hospital, Nottingham, United Kingdom; 3 Nottingham Respiratory Research Unit, University of Nottingham, Nottingham, United Kingdom; 4 Health and Social Care Information Centre, Leeds, United Kingdom; University of Nebraska Medical Center, United States of America

## Abstract

**Background:**

The purpose of this study was to identify trends in survival and chemotherapy use for individuals with small-cell lung cancer (SCLC) in England using the National Lung Cancer Audit (NLCA).

**Methods:**

We used data from the NLCA database to identify people with histologically proven SCLC from 2004–2011. We calculated the median survival by stage and assessed whether patient characteristics changed over time. We also assessed whether the proportion of patients with records of chemotherapy and/or radiotherapy changed over time.

**Results:**

18,513 patients were diagnosed with SCLC in our cohort. The median survival was 6 months for all patients, 1 year for those with limited stage and 4 months for extensive stage. 69% received chemotherapy and this proportion changed very slightly over time (test for trends p = 0.055). Age and performance status of patients remained stable over the study period, but the proportion of patients staged increased (p-value<0.001), mainly because of improved data completeness. There has been an increase in the proportion of patients that had a record of receiving both chemotherapy and radiotherapy each year (from 19% to 40% in limited and from 9% to 21% in extensive stage from 2004 to 2011). Patients who received chemotherapy with radiotherapy had better survival compared with any other treatment (HR 0.24, 95% CI 0.23–0.25).

**Conclusion:**

Since 2004, when the NLCA was established, the proportion of patients with SCLC having chemotherapy has remained static. We have found an upward trend in the proportion of patients receiving both chemotherapy and radiotherapy which corresponded to a better survival in this group, but as it only applied for a small proportion of patients, it was not enough to change the overall survival.

## Introduction

Small-cell lung cancer (SCLC) accounted for 20% of all lung cancer cases diagnosed over a decade ago [1] but this proportion has decreased and currently only accounts for approximately 10%. [2], [3], [4], [5] SCLC is responsive to chemotherapy [6] (and combination chemo-radiotherapy) [7] and this is the main treatment recommended by the National Institute of Health and Clinical Excellence (NICE). [3] However, despite the sometimes dramatic response to chemotherapy, many patients relapse and die within 6 months of diagnosis. [8] Furthermore, survival from SCLC is poor in England compared with other European and North American countries, [9], [10], [11] with only 5% of the patients surviving for at least 5 years. [12].

The National Lung Cancer Audit (NLCA) was established in 2004 to measure the outcomes and quality of care for patients with lung cancer provided by the National Health Services (NHS) and in doing so to improve the quality of the service. [13] The audit has been used to set standards of care, such as 80% of patients should be seen by lung cancer nurse specialists, 75% of patients should have histological confirmation, and 95% patients should be discussed by a multi-disciplinary team (MDT). These standards are designed to make the treatment and care given in England more comparable to other European and North American countries. We used the NLCA database linked with the Hospital Episode Statistic (HES) database to assess the impact of NLCA on the English lung cancer population by studying the trends in chemotherapy use, survival and changing features of patients with SCLC since the audits introduction in 2004.

## Methods

### Data Source and Study Population

The NLCA database is a longitudinal database consisting of anonymous computerised records of individuals with a diagnosis of primary lung cancer. It has collected data on demographics, tumour features and treatment since 2004 via 157 English NHS hospitals responsible for managing and treating patients with lung cancer. Data is usually entered by members of lung cancer MDT. Using the NLCA database, we identified all English cases with histologically proven SCLC diagnosed between 1^st^ January 2004 and 31^st^ December 2011. The NLCA dataset has been analysed previously as part of a validation process [13], and currently the case ascertainment is in excess of 90% [4], [5]. We used linked data from Hospital Episode Statistics (HES), a mandatory national database collecting data on all in-patient diagnoses, consultant referrals and treatment procedure performed, to provide additional information on co-morbidity and treatment and the Office for National Statistics (ONS) which collects data from death certificates, the registration of which is a legal requirement in the United Kingdom (UK).

### Covariates

For this study, we restricted our analyses to those patients with a histologically proven diagnosis of SCLC based on the recorded Systematised Nomenclature of Medicine (SNOMED) codes in the NLCA database (M-8041/3). Our initial dataset included information on age at diagnosis, sex, performance status (PS) and stage. PS was classified according to the World Health Organisation (WHO) definition and the stage was recorded using the Veteran’s Administration Lung Study Group system (limited or extensive). For a few cases where the most recent Tumour Node Metastases staging system (TNM) was used, we converted this to limited (T1-4, N0-3, M0) or extensive (M1a/b) as appropriate. [14] We defined socio-economic status (SES) using the Townsend deprivation Index, which uses a composite score of four variables (unemployment, overcrowding, non-car ownership and non-home ownership), and is split into 5 categories of deprivation. However due to more than 90% of missing data on SES from 2004–2005, we performed a separate analysis for 2006–2011.

To determine overall survival, we created a start date which was the date of diagnosis. In the absence of a date of diagnosis, a pseudo start date was generated using the median number of days (for the whole cohort) between date of diagnosis and the following dates in this order: (1) date first seen, (2) date of referral, or (3) date discussed by MDT. An end date for each patient was created using either the date of death (provided by ONS) or the date of the last ONS cross-check for death dates (31^st^ March 2013). Therefore every patient had a minimum of 15 months of follow up for the survival analysis.

### Chemotherapy and Radiotherapy

We used the NLCA and HES dataset to determine whether an individual in our cohort had received chemotherapy. From HES in-patient hospital episodes for each patient, we used International Classification of Diseases (ICD) codes Z51.1 & Z51.2 allocated for ‘chemotherapy session for neoplasm’ and ‘other chemotherapy’ to indicate chemotherapy provision. We also identified Office of Population Censuses and Survey Classification of Intervention (OPCS-4) codes for chemotherapy from the HES database. Presence of either one or both of the ICD/OPCS-4 codes from the HES database was taken as evidence of receiving chemotherapy. We also identified patients who had chemotherapy from the NLCA database as HES does not record chemotherapy given during an out-patient admission. Patients were classified as not receiving chemotherapy if there was no date of chemotherapy in the NLCA or HES. Patients were included in our study if their date of diagnosis was in our study period. We excluded patients from our cohort who had received their first dose of chemotherapy 1 month prior or 6 months after the date of diagnosis of lung cancer. This step was done to minimise the skewing of overall survival time in either direction by excluding patients who may have received chemotherapy for some other cancer prior to being diagnosed with SCLC or received chemotherapy for a slow growing cancer which had been misclassified as SCLC (6 months after date of diagnosis).

As the NLCA does not collect detailed information on radiotherapy treatment type and intent, it is difficult to know whether the radiotherapy was given for curative or palliative purpose. However, we used the NLCA database to identify patients who received radiotherapy using the date of radiotherapy given. It was also difficult to identify patients who received concurrent chemotherapy and radiotherapy as the NLCA records only the first dose of either treatment. Only 404 patients (2%) had clear evidence of concurrent chemotherapy and radiotherapy.

### Statistical Analysis

All data management and statistical analyses were performed using Stata version 12 (StataCorp, Texas). Initially, we calculated the median age of diagnosis and median survival in days by the year in which a patient was diagnosed with SCLC. The first two years, 2004 and 2005, were grouped together to create a comparator group of adequate size. We also looked at the patient features at the time of diagnosis by year and performed the Cuzick’s non-parametric test. A p-value of <0.05 was considered as significant. We also looked at the proportion of patients receiving chemotherapy and the proportion who received both chemotherapy and radiotherapy. We performed Cox regression analysis to calculate the hazard ratios (HRs) depending on the type of treatment received after the diagnosis compared with no treatment received after diagnosis of SCLC, adjusted for patient features and years.

### Ethics

The data was obtained from the Heathcare Quality Improvement Partnership (HQIP). Ethical approval from the University of Nottingham medical school research ethnics committee was obtained by the researchers to work on a linked HES and NLCA dataset (RU943 177570-MV6J3). The NLCA has Ethics and Confidentiality Committee (ECC) approval to use patient information from the National Health Services (NHS). Finally for this specific set of work, we also obtained approval from HQIP who commission the audit and HSCIC caldicott guardian signed off the data sharing agreement [IG Reference: IC381DS]. The data was anonymised in the linked dataset by the HSCIC personal prior to be given to the researchers.

## Results

There were a total of 178,427 individuals diagnosed with lung cancer in the NLCA between 1^st^ January 2004 and 31^st^ December 2011. We restricted our analyses to only those individuals who had a histologically proven SCLC (n = 18,513 (10.3%)). Table 1 shows the patient features by year of diagnosis.

**Table 1:** Changing features of patients with small-cell lung cancer over the duration of the NLCA (n = 18,513).

	Year of Diagnosis							
	2004/2005	2006	2007	2008	2009	2010	2011	test for trends
**Number of patients N**	1867	1836	2081	2697	3208	3325	3499	
**Median Survival in days**								
Whole cohort (n = 18513)	179	179	190	190	179	186	190	
Limited stage (n = 4830)	358	321	343	362	332	358	339	
Extensive stage (n = 9874)	102	120	139	124	113	124	124	
Stage Unknown (n = 772)	168	135	183	219	128	223	256	
Stage uncertain (n = 933)	226	208	208	194	237	201	285	
Stage missing (2104)	168	164	164	161	142	142	168	
**Median age at diagnosis**	67.9	68.2	68.3	68.1	68.3	68.6	68.7	
**Sex (%)**								
Female	879 (47.08)	843 (45.92)	926 (44.50)	1248 (46.27)	1537 (47.91)	1616 (48.60)	1741 (49.76)	
Male	988 (52.92)	993 (54.08)	1155 (55.50)	1449 (53.73)	1671 (52.09)	1709 (51.40)	1758 (50.24)	<0.001
**Age n (%)**								
<65	668 (35.78)	634 (34.33)	709 (34.07)	940 (34.85)	1105 (34.45)	1121 (33.71)	1144 (32.70)	
65–75	607 (32.51)	612 (33.33)	710 (34.12)	901 (33.41)	1103 (34.38)	1124 (33.80)	1219 (34.84)	
>75	592 (31.71)	590 (32.14)	662 (31.81)	856 (31.74)	1000 (31.17)	1080 (32.48)	1136 (32.47)	0.101†
**Performance Status (%)**								
0	223 (11.94)	252 (13.73)	261 (12.54)	338 (12.53)	456 (14.21)	503 (15.13)	550 (15.72)	
1	395 (21.16)	454 (24.73)	533 (25.61)	746 (27.66)	948 (29.55)	1039 (31.25)	1229 (35.12)	
2	304 (16.28)	327 (17.81)	411 (19.75)	506 (18.76)	648 (20.20)	736 (22.14)	747 (21.35)	
3	188 (10.07)	182 (9.91)	221 (10.62)	351 (13.01)	478 (14.90)	480 (14.44)	510 (14.58)	
4	67 (3.59)	60 (3.27)	78 (3.75)	91 (3.37)	132 (4.11)	148 (4.45)	152 (4.34)	0.877†
missing	690 (36.96)	561 (30.56)	577 (27.73)	665 (24.66)	546 (17.02)	419 (12.60)	311 (8.89)	
**Charlson Index (%)**								
0	815 (43.65)	736 (40.09)	811 (38.97)	912 (33.82)	1035 (32.26)	954 (28.69)	948 (27.09)	
1	321 (17.19)	344 (18.74)	383 (18.40)	486 (18.02)	581 (18.11)	595 (17.89)	603 (17.23)	
2–3	191 (10.23)	218 (11.87)	238 (11.44)	346 (12.83)	415 (12.84)	436 (13.11)	471 (13.46)	
4+	540 (28.92)	538 (29.30)	649 (31.19)	953 (35.34)	1177 (36.69)	1340 (40.30)	1477 (42.21)	<0.001†
**Stage (%)**								
SCLC-Limited	449 (24.05)	451 (24.56)	480 (23.07)	643 (23.84)	893 (27.84)	887 (26.68)	1027 (29.35)	
SCLC-Extensive	794 (42.53)	824 (44.88)	951 (45.70)	1356 (50.28)	1693 (52.77)	2020 (60.75)	2236 (63.90)	
SCLC-Unknown	177 (9.48)	246 (13.40)	156 (7.50)	90 (3.34)	62 (1.93)	24 (0.72)	17 (0.49)	
SCLC-Uncertain	37 (1.98)	57 (3.10)	245 (11.77)	285 (10.57)	193 (6.02)	93 (2.80)	23 (0.66)	
Stage missing	410 (21.96)	258 (14.05)	249 (11.97)	323 (11.98)	367 (11.44)	301 (9.05)	196 (5.60)	<0.001†
**Chemotherapy n (%)**	1236 (66.20)	1266 (68.95)	1483 (71.26)	1878 (69.63)	2188 (68.20)	2282 (68.63)	2478 (70.82)	0.055
**Socio-economic status (%)**								
1 (Most Affluent)		234 (12.75)	314 (15.09)	395 (14.65)	494 (15.40)	458 (13.77)	487 (13.92)	
2		312 (16.99)	383 (18.40)	469 (17.39)	582 (18.14)	600 (18.05)	657 (18.78)	
3		348 (18.95)	413 (19.85)	546 (20.24)	620 (19.33)	615 (18.50)	716 (20.46)	
4		381 (20.75)	455 (21.86)	575 (21.32)	738 (23.00)	711 (21.38)	789 (22.55)	
5 (Least Affluent)		499 (27.18)	515 (24.75)	698 (25.88)	722 (24.06)	753 (22.65)	817 (23.35)	0.015†
Missing		62 (3.38)	1 (0.05)	14 (0.52)	2 (0.06)	188 (5.65)	33 (0.94)	

† Cuzick’s non-parametric test for trends otherwise chi-square test for trends.

The median age at diagnosis remained the same at 69 years (interquartile range (IQR) 62–75) from 2004–2011. A total of 12,811 (69.2%) patients received chemotherapy in our cohort and this proportion had increased very slightly over the study period showing a borderline significant trend (test for trends 0.055). Age and PS did not change over the years. There was a significant change in recording of stage (test for trends <0.001), with a decreasing proportion of patients with unknown, uncertain and missing stage and increasing proportions of limited and extensive stage SCLC. There was also a significant change in the distribution of co-morbidity over the years, with more patients having a Charlson index of 4 or more in more recent years (test for trend across Charlson score groups p<0.001).

Table 2 represents the multivariate logistic regression analyses for patients diagnosed between 2006 and 2011 (2004/2005 excluded because of high level of missing data on socioeconomic status). The odds of receiving chemotherapy reduced with increasing age, PS and co-morbidity (χ2 p-value for trends <0.001). We also observed a significant association between receiving chemotherapy and SES (p_trends_<0.001), where patients from least affluent areas were 13% less likely to receive chemotherapy compared with patients from the most affluent areas after adjusting for confounders (adjusted OR 0.87, 95% CI 0.77–0.99).

**Table 2:** Results for multivariate logistic regression analysis for patients diagnosed between 2006–2011 (n = 16,646).

	Number ofpatients n (%)	Proportion of patientswho receivedchemotherapy n (%)	Unadjusted OR(95% CI)	Adjusted OR(95% CI)‡	p-value
**Sex**					
Female	7911 (48)	5533 (70)	1	1	
Male	8735 (52)	6042 (69)	0.96 (0.90–1.03)	0.98 (0.91–1.06)	0.74†
**Age**					
<65	5653 (34)	4670 (83)	1	1	
65–75	5669 (34)	4170 (74)	0.58 (0.53–0.64)	0.64 (0.58–0.71)	
>75	5324 (32)	2735 (51)	0.22 (0.20–0.24)	0.25 (0.23–0.28)	<0.001
**Performance Status**					
0	2360 (14)	2107 (89)	1	1	
1	4949 (30)	4220 (85)	0.69 (0.59–0.80)	0.88 (0.75–1.03)	
2	3375 (20)	2289 (68)	0.25 (0.21–0.29)	0.39 (0.33–0.46)	
3	2222 (13)	899 (40)	0.08 (0.06–0.09)	0.13 (0.11–0.15)	
4	661 (4)	80 (12)	0.01 (0.01–0.02)	0.02 (0.02–0.03)	<0.001
missing	3079 (19)	1980 (64)	0.21 (0.18–0.25)	0.30 (0.25–0.35)	
**Stage**					
SCLC-Limited	4381 (26)	3522 (80)	1	1	
SCLC-Extensive	9080 (55)	5952 (66)	0.46 (0.42–0.50)	0.67 (0.60–0.74)	
SCLC-others	3185 (19)	2101 (66)	0.47 (0.42–0.52)	0.65 (0.58–0.74)	<0.001†
**Charlson Index**					
0	5396 (32)	4363 (81)	1	1	
1	2992 (18)	2211 (74)	0.67 (0.60–0.74)	0.81 (0.72–0.92)	
2–3	2124 (13)	1432 (67)	0.48 (0.43–0.54)	0.67 (0.59–0.74)	
4+	6134 (37)	3569 (58)	0.32 (0.30–0.35)	0.50 (0.45–0.55)	<0.001
**Socio-economic status**					
1 (Most affluent)	2382 (14)	1664 (70)	1	1	
2	3003 (18)	2133 (71)	1.05 (0.94–1.19)	1.00 (0.87–1.15)	
3	3258 (20)	2278 (70)	1.00 (0.89–1.12)	0.99 (0.86–1.13)	
4	3649 (22)	2526 (69)	0.97 (0.86–1.08)	0.84 (0.74–0.96)	
5	4054 (24)	2828 (70)	0.99 (0.89–1.11)	0.87 (0.77–0.99)	0.001
Missing	300 (2)	146 (49)	0.40 (0.32–0.52)	0.44 (0.33–0.59)	

‡Adjusted for all other variables in the table.

†Log-likelihood ratio p-value - otherwise χ2 p-value.

### Chemotherapy and Radiotherapy

Table 3 shows the proportion of SCLC patients receiving chemotherapy alone, radiotherapy alone and chemotherapy and radiotherapy, stratified by stage. The proportion of patients who received chemotherapy remained stable for all stages over the years; however there was an increase in the proportion of patients with recorded chemotherapy and radiotherapy for all stages. In limited stage it increased from 19% to 40% and in extensive stage from 10% to 21%. There was also an increase of recorded chemotherapy and radiotherapy for unknown, uncertain and missing stage. The use of radiotherapy alone, regardless of stage, had also increased over the years. Patients who received chemo-radiotherapy had a better median survival (335 days) compared with chemotherapy alone (235 days), radiotherapy alone (82 days) and no therapy (24 days) (data not shown). It was also observed that patients who received chemo-radiotherapy were younger, with less co-morbidity and had better PS compared with patients who received chemotherapy or radiotherapy alone (table S1).

**Table 3:** Proportion of people receiving chemotherapy and radiotherapy by stage (n = 18513).

Year	Total patientsreported n (%)	Total patients withrecorded chemotherapyn (%)	Patients with recordedchemotherapy + radiotherapy n (%)	Recorded radiotherapy only n (%)
**Limited stage small-cell (n = 4830)**				
2004/2005	449	363 (80.85)	87 (19.38)	16 (3.56)
2006	451	356 (78.94)	50 (11.09)	15 (3.33)
2007	480	387 (80.63)	88 (18.33)	26 (5.42)
2008	643	501 (77.92)	172 (26.75)	37 (5.75)
2009	893	714 (79.96)	331 (37.07	52 (5.82)
2010	887	714 (80.50)	332 (37.43)	46 (5.19)
2011	1027	850 (82.77)	411 (40.02)	43 (4.19)
**Extensive stage small-cell (n = 9874)**				
2004/2005	794	481 (60.58)	77 (9.70)	40 (5.04)
2006	824	548 (66.50)	69 (8.37)	43 (5.22)
2007	951	638 (67.09)	102 (10.73)	44 (4.63)
2008	1356	910 (67.11)	217 (16.00)	86 (6.34)
2009	1693	1068 (63.08)	292 (17.25)	139 (8.21)
2010	2020	1311 (64.90)	424 (20.99)	141 (6.98)
2011	2236	1477 (66.06)	474 (21.20)	129 (5.77)
**Unknown stage small-cell (n = 772)**				
2004/2005	177	108 (61.02)	22 (12.43)	11 (6.21)
2006	246	164 (66.67)	19 (7.72)	13 (5.28)
2007	156	111 (71.15)	28 (17.95)	4 (2.56)
2008	90	65 (72.22)	14 (15.56)	4 (4.44)
2009	62	42 (67.74)	15 (24.19)	5 (8.06)
2010	24	14 (58.33)	2 (8.33)	3 (12.50)
2011	17	12 (70.59)	4 (23.53)	0 (0.00)
**Uncertain stage (n = 922)**				
2004/2005	37	23 (62.16)	4 (10.81)	2 (5.41)
2006	57	36 (63.16)	5 (8.77)	1 (1.75)
2007	245	178 (72.65)	19 (7.76)	14 (5.71)
2008	285	190 (66.67)	62 (21.75)	22 (7.72)
2009	193	139 (72.02)	50 (25.91)	11 (5.70)
2010	93	62 (66.67)	20 (21.51)	6 (6.45)
2011	23	15 (65.22)	5 (21.74)	2 (8.70)
**Stage missing (n = 2104)**				
2004/2005	410	261 (63.66)	25 (6.10)	18 (4.39)
2006	258	162 (62.79)	17 (6.59)	12 (4.65)
2007	249	169 (67.87)	21 (8.43)	7 (2.81)
2008	323	212 (65.63)	50 (15.48)	20 (6.19)
2009	367	225 (61.31)	60 (16.35)	20 (5.45)
2010	301	181 (60.13)	58 (19.27)	22 (7.31)
2011	196	124 (63.40)	45 (22.96)	12 (6.12)

### Survival

**Figure 1:**
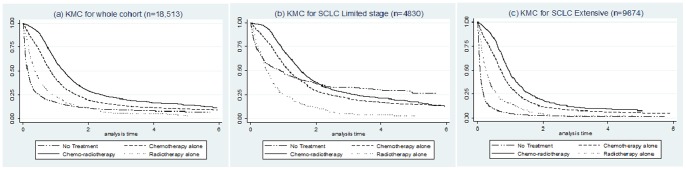
Kaplan-Meier curve estimates adjusted for age, sex, PS and co-morbidity by treatments received: (a) for whole cohort, (b) for SCLC limited and (c) for SCLC Extensive.

Median survival (MS) from the time of diagnosis for all the patients (N = 18,513) was 6 months (IQR 1.5–12.4). This was 11.4 months (5.5–21.1) and 4 months (1.0–9.0) for patients with limited and extensive stage disease (table 1). Kaplan-Meier survival curves (Figure 1) also showed a difference in overall hazard ratios (HR) based on the treatment received having adjusted for patient features. Compared with patients who received no treatment (MS = 0.72 months), patients who received chemotherapy had an adjusted HR of 0.33, 95% CI 0.32–0.34 (MS = 7.6 months) and patients who had records of radiotherapy and chemotherapy had an adjusted HR of 0.24, 95% CI 0.23–0.25 (MS = 11.6 months). We also looked at the hazard ratios by limited and extensive stage which showed a similar improved survival from receiving any treatment compared with no treatment. However in limited stage disease, there was only a small difference in survival for patients receiving no treatment and those having radiotherapy alone.

## Discussion

### Principal Findings

Histologically proven SCLC accounted for 10% of the total lung cancer cases diagnosed in the NLCA between 2004 and 2011. Survival in SCLC is poor and our results demonstrate that the median survival has not changed in the 8 year period since the audit began. The proportion of patients receiving chemotherapy has increased very slightly over the years and although there has been an increase in the proportion of patients receiving chemotherapy and radiotherapy we observed no change in the overall survival. One of the reasons for this could be the increase in radiotherapy and chemo-radiotherapy observed in our study was actually due to an increased use of prophylactic cranial irradiation (PCI). This treatment is recommended in the NICE (2011) updated guidelines [3], and has shown survival benefits in studies. [15] However, it could not change our survival estimates as the doubling proportion only accounted for a 20% increase in chemo-radiotherapy from 2004–2011, which is still lower than other comparable countries. In our study, there has been little change in the patient demographics from 2004–2011. However, there has been an increase in the proportions classified as extensive and limited stage small-cell lung cancer and an increase in the proportion of patients with more co-morbid illness, which may lead to fewer patients being considered for curative treatment. There was also inequality in chemotherapy use by socioeconomic status where patients from least affluent areas were less likely to receive chemotherapy.

Our results depict a low proportion of patients receiving chemo-radiotherapy, especially for limited stage SCLC. However our results are a reflection of the true treatment patterns and attitude towards treatment in England. It should be noted that even patients with limited stage SCLC can be considered too frail to receive chemo-radiotherapy and in our cohort, one-third of the limited stage SCLC patients had a PS of greater than or equal to 2 and almost the same proportion had a Charlson Index of greater than or equal to 2.

Our results showed that patients who had recorded chemotherapy and radiotherapy had a longer median survival and lower hazard ratio of death, which may reflect treatment efficiency or selection bias such as immortal time bias. Immortal time bias here refers to the fact that some patients, with an inherently better prognosis would receive treatment whereas those with a poor prognosis (associated with more advance disease and co-morbidity) would not. The treatment may be having no effect on prognosis and the apparent better survival may be simply a result of more favourable biological factors.

### Strengths and Limitations

The main strength of our study is the large sample size. As far as we know, this is the largest study looking at the trends in chemotherapy use and features of patients with SCLC in England over the duration of the NLCA. Although the NLCA is non-mandatory, it has been validated and found to be representative of the population of lung cancer patients in England. [13] The NLCA provides more data compared with databases used by other international studies (in which case-mix adjustment is not possible). [9] Therefore we believe our results are likely to demonstrate real changes in chemotherapy practice in England. The cases identified in our study were all pathologically confirmed. Within the NLCA missing or unknown pathology is coded as non-small cell lung cancer. Thus some of these cases could have been small cell lung cancer. It is unlikely that this is a significant proportion and most unlikely that this would affect our conclusions. The proportion of small-cell cases observed in our study is in accordance with a study using the Thames Cancer Registry data. [2] A second limitation is the high proportion of patients with stage missing, uncertain and unknown, although this is decreasing year on year. We did not find any evidence of a change in the ratio of limited to extensive stage disease which suggests that the stage distribution did not change of the years and therefore the comparisons of survival by stage over the years was valid. It is unlikely that those patients with missing or uncertain stage were misclassified because of poorer prognosis, as we found their survival was better than those with extensive disease.

We were able to validate records of chemotherapy by using a combination of variables from NLCA and HES, but as inpatient HES data does not capture the majority of radiotherapy episodes, we might have underestimated the total proportion of patients who had radiotherapy. In addition, due to the limited data on radiotherapy, we were unable identify whether the radiotherapy was given with curative or palliative intent.

We have shown an increase in the proportion of patients receiving both chemotherapy and radiotherapy however our data were unable to differentiate radical intent chemo-radiotherapy from radiotherapy given for purely palliative purposes. Chemo-radiotherapy is associated with fairly big survival benefits in clinical trials and in our study there was some evidence of this. However, our group receiving chemotherapy and radiotherapy are likely to include a significant proportion of patients who had both for palliative purposes and therefore any survival benefits of chemo-radiotherapy would have been diluted. This probably explains why the overall survival did not change with the increase in the proportion of patients receiving chemotherapy and radiotherapy.

### Comparison with other Studies

We used a combination of databases and definitions to identify patients who had received chemotherapy on an in-patient and out-patient basis, and found that almost 70% of our cohort had received chemotherapy which was 10% higher than an earlier study based on the NLCA database. [8] Previous studies including one study conducted in the Netherlands [16] and one study using the Surveillance Epidemiologic and End Results (SEERs) database [17] showed survival improvement for patients with SCLC, which we did not observe. However, the findings of the SEERs study of a 3% annual change in survival in both limited and extensive stage SCLC were over a longer timeframe (1973–2002) than in the present study. [17] Over this period treatment is likely to have changed much more than from 2004 to 2011. Chemotherapy regimens, supportive care and the use of combined chemo-radiotherapy have changed to a greater extent based on evidence from clinical trial in 1987 [18] and multiple meta-analysis in the 1990s [19], [20] showing chemo-radiotherapy to have better outcomes to chemotherapy alone promoting the use of newer therapies. To our knowledge, there have been no new changes in the treatment regimen of SCLC since 2004 and this is the likely explanation for the lack of an annual overall survival improvement in our study. Our study shows the inequality in chemotherapy use in older patients (i.e. 65–75 & >75 years) even after adjusting for several other patient features, which is similar to the results found by other studies. [8], [21], [22] A similar association was seen in patients from more deprived areas who were less likely to receive chemotherapy, which is similar to the findings of other studies. [8], [23] We also observed that the proportion of patients with comorbidities increased over the years, which is not seen in previous studies. The reason for this is unknown but could be due to better recording of comorbidities in the HES database.

### Clinical Relevance

The overall survival of patients with SCLC has not changed since the audit initiated in 2004, probably due to the fact that there has been little change in the type of treatment offered or in the stage at presentation. The only improvement we observed over the years was an increase in the proportion of patients who received radiotherapy in addition to chemotherapy which corresponded to a better survival compared with other treatment regimens but as this applied to only small proportion of the patients as a whole, it was not enough to change the overall survival. The reasons for the unchanged survival are unknown but this could be due to the relatively modest advances in treatment for a small proportion of patients (mostly chemo-radiotherapy for limited stage). It is concerning that only a relatively small proportion of patients receive chomo-radiotherapy, as this is the established best standard of care, and clearly associated with better survival. The therapy does not require patients to have a reasonable level of fitness and co-morbidity rates in the UK can be high. Nevertheless it is important for clinicians to reflect on our findings and to consider whether more patients might be offered this apparently more effective treatment.

The UK radiotherapy database and Systemic Anti-cancer Therapy dataset (SACT) started to collect data in April 2009 and April 2012, [24] and in due course, analysis of these data would provide us with a more detailed picture of the impact of increasing radiotherapy use on survival for patients with SCLC.

## Supporting Information

Table S1 **Baseline characteristics of patients receiving chemo-radiotherapy, chemotherapy alone or radiotherapy alone (n = 18,513).**
(DOCX)
