# Supplementary material for: Small-Cell Lung Cancer in England: Trends in Survival and Chemotherapy Using the National Lung Cancer Audit
Source: PLoS One. 2014 Feb 21;9(2):e89426. doi: 10.1371/journal.pone.0089426 (PMC3931780; doi:10.1371/journal.pone.0089426)
Supplement: Table S1 — Baseline characteristics of patients receiving chemo-radiotherapy, chemotherapy alone or radiotherapy alone (n = 18,513). (DOCX) [file pone.0089426.s001.docx]

**Table S1:** Baseline characteristics of patients receiving chemo-radiotherapy, chemotherapy alone or radiotherapy alone (n=18,513)

|  | **Number of patients n (%)** | | |
| --- | --- | --- | --- |
|  | **Chemo-radiotherapy** | **Chemotherapy alone** | **Radiotherapy alone** |
| **Sex** |  |  |  |
| Female | 1792 (49) | 4341 (47) | 525 (49) |
| Male | 1879 (51) | 4799 (53) | 541 (51) |
| **Age** |  |  |  |
| <65 | 1655 (45) | 3536 (39) | 225 (21) |
| 65-75 | 1302 (36) | 3295 (36) | 283 (27) |
| >75 | 714 (19) | 2309 (25) | 558 (52) |
| **Performance status** | |  |  |
| 0 | 807 (22) | 1489 (16) | 53 (5) |
| 1 | 1472 (40) | 3080 (34) | 210 (20) |
| 2+ | 869 (24) | 2700 (30) | 578 (54) |
| Missing | 523 (14) | 1871 (20) | 225 (21) |
| **Charlson Index** | |  |  |
| 0 | 1481 (40) | 3509 (38) | 263 (25) |
| 1 | 733 (20) | 1698 (19) | 177 (17) |
| 2-3 | 425 (12) | 1120 (12) | 143 (13) |
| 4+ | 1032 (28) | 2813 (31) | 483 (45) |
